# Upregulated ENC1 predicts unfavorable prognosis and correlates with immune infiltration in endometrial cancer

**DOI:** 10.3389/fcell.2022.919637

**Published:** 2022-12-01

**Authors:** Lingling He, Wenjing He, Ji Luo, Minjuan Xu

**Affiliations:** ^1^ Department of Obstetrics and Gynecology, Ganzhou People's Hospital, Ganzhou, China; ^2^ Department of Obstetrics and Gynecology, Ganzhou Hospital-Nanfang Hospital, Southern Medical University, Ganzhou, China; ^3^ Department of Obstetrics and Gynecology, The Affiliated Ganzhou Hospital of Nanchang University, Ganzhou, China; ^4^ Department of Endocrinology, Baoji Gaoxin Hospital, Baoji, China

**Keywords:** endometrial carcinoma, ENC1, WGCNA, prognosis, immunity therapy

## Abstract

A better knowledge of the molecular process behind uterine corpus endometrial carcinoma (UCEC) is important for prognosis prediction and the development of innovative targeted gene therapies. The purpose of this research is to discover critical genes associated with UCEC. We analyzed the gene expression profiles of TCGA-UCEC and GSE17025, respectively, using Weighted Gene Co-expression Network Analysis (WGCNA) and differential gene expression analysis. From four sets of findings, a total of 95 overlapping genes were retrieved. On the 95 overlapping genes, KEGG pathway and GO enrichment analysis were conducted. Then, we mapped the PPI network of 95 overlapping genes using the STRING database. Twenty hub genes were evaluated using the Cytohubba plugin, including NR3C1, ATF3, KLF15, THRA, NR4A1, FOSB, PER3, HLF, NTRK3, EGR3, MAPK13, ARNTL2, PKM2, SCD, EIF5A, ADHFE1, RERGL, TUB, and ENC1. The expression levels of NR3C1, PKM2, and ENC1 were shown to be adversely linked with the survival time of UCEC patients using univariate Cox regression analysis and Kaplan-Meier survival calculation. ENC1 were also overexpressed in UCEC tumor tissues or cell lines, as shown by quantitative real-time PCR and Western blotting. Then we looked into it further and discovered that ENC1 expression was linked to tumor microenvironment and predicted various immunological checkpoints. In conclusion, our data indicate that ENC1 may be required for the development of UCEC and may serve as a future biomarker for diagnosis and therapy.

## Introduction

Uterine corpus endometrial carcinoma (UCEC) is one of the most common gynecologic cancers with a global prevalence that is rising (Siegel et al., 2021). Obesity is a major risk factor for UCEC, and as obesity rises, the number of cases is anticipated to double by 2030 (Popli et al., 2020). In recent years, as a result of changing lifestyles and increased use of estrogen replacement therapy, the prevalence of UCEC has increased dramatically, posing a severe danger to women’s health (Zhu et al., 2020). At the moment, surgery is the primary method of UCEC, although suitable adjuvant therapy is also used, depending on the pathological and clinical phases of the tumors. However, about one-fifth of cancers come back after surgery, and systematic therapy doesn’t work very well (Blake et al., 2009). The absence of early identification and therapy to halt carcinogenesis or progression has been blamed for the majority of UCEC fatalities. Thus, it is critical to identify appropriate biomarkers and prospective targets for the correct prediction or diagnosis of UCEC, as well as to investigate ways to enhance the treatment impact and clinical prognosis of UCEC patients.

With the advancement of genomic technology, bioinformatics has grown in popularity for analyzing gene expression patterns in order to better understand the molecular processes behind illnesses and to identify meaningful therapeutic targets (Cantelli et al., 2022; Verga et al., 2022). Weighted Gene Co-expression Network Analysis (WGCNA) is a critical technique for deciphering gene function and association from genome-wide expression (Liang et al., 2022; Rezaei et al., 2022). Additionally, a strong technique within transcriptomics is differential gene expression analysis, which enables the investigation of the molecular processes behind genome regulation and the discovery of quantifiable differences in expression levels between experimental and control groups (Hu et al., 2021; Hou et al., 2022). Thus, by combining the results of WGCNA and differential gene expression analysis, the discriminating ability of highly linked genes valuable as potential biomarkers is enhanced (Bai et al., 2020).

The transcriptional data for UCEC were evaluated in this work using WGCNA and differential gene expression analysis to identify differentially expressed genes. We investigated the development of UCEC in further detail using functional enrichment and protein-protein interaction (PPI) analysis in conjunction with survival analysis. By assessing differential co-expression genes for clinical diagnosis or therapy, the research establishes a viable foundation for understanding the etiology and probable molecular processes of UCEC.

## Materials and methods

### Data set acquisition

We obtained an RNA-sequencing dataset from The Cancer Genome Atlas (TCGA, https://portal.gdc.cancer.gov/) consisting of 23 normal endometrium tissue samples and 552 endometrial cancer cases with associated clinical data. Additionally, 12 normal endometrial samples and 91 endometrial cancer samples with prognostic information were retrieved from the Gene Expression Omnibus (GEO) dataset (https://www.ncbi.nlm.nih.gov/geo/). As stated in the tutorial for the edgeR package, genes with low read counts often do not need further analysis (Robinson et al., 2010). As a result, we retained genes with a CPM (count per million) greater than one in our research. The analysis was carried out using the Limma program (Ritchie et al., 2015). The Benjamini-Hochberg approach was used to alter the *p*-value in order to maintain a low false discovery rate (FDR) (Ghosh, 2019). Additionally, we utilized the Limma program to identify genes that were differently expressed when |log2 fold change (FC)| ≥ 1 and false discovery rate (FDR) < 0.05 were employed.

### Identifying modules using weighted gene co-expression network analysis

The WGCNA package in R was used to create gene co-expression networks using the gene expression profiles of TCGA-UCEC and Gene Expression Omnibus Series 17,025 (GSE 17025) in our research (Langfelder and Horvath, 2008). WGCNA was used to look at the modules of highly correlated genes in different samples so that relationships between modules and sample characteristics could be found. Following that, we generated an adjacency matrix and converted it to TOM (Shuai et al., 2021). To aid in the identification of functional modules within a co-expression network, module-trait connections between modules were generated using previously published data (Li et al., 2020). As a result, modules with a high correlation coefficient were chosen for further investigation as candidates for clinical features. Following that, we examined the overlapping genes between DEGs and co-expression genes to find possible prognostic genes, which were visualized using the R program VennDiagram (Chen and Boutros, 2011).

### Functional enrichment analysis and establishment of a protein-protein interaction network

In this work, the R program “clusterProfiler” was used to evaluate the biological function of the genes (Yu et al., 2012). The R package “ggplot2” was used to visualize Gene Ontology (GO) enrichment and Kyoto Encyclopedia of Genes and Genomes (KEGG) pathways (Steenwyk and Rokas, 2021). In this research, we constructed a protein–protein interactions (PPI) network of chosen genes using the STRING web tool (Szklarczyk et al., 2019). Genes with a score of 0.7 were picked from the STRING database to create a network model shown in Cytoscape (Doncheva et al., 2019). The Maximal Clique Centrality (MCC) algorithm, calculated by CytoHubba (Chin et al., 2014), was shown to be the most effective technique for locating hub nodes in a co-expression network. The genes with the top 20 MCC scores were deemed to be hub genes in this research.

### Assessing transcript levels and prognostic value of hub genes

Using data from the TCGA database, a Kaplan–Meier univariate survival analysis was used to investigate the connection between overall survival (OS) and hub genes in patients. In our research, we chose for survival analysis those patients who had finished their follow-up periods and then separated them into two distinct groups based on the median expression value of hub genes. A *p*-value less than 0.05 was considered statistically significant. Additionally, we obtained the transcription levels of hub genes in UCEC patients using the online tool GEPIA2 (Tang et al., 2019)(http://gepia2.cancer-pku.cn/#index).

### Tumor immune estimation resource database analysis

To assess the relationships between the levels of immune cell infiltration and ENC1 expression, we employed the Tumor Immune Estimation Resource (TIMER; https://cistrome.shinyapps.io/timer/). Additionally, Spearman’s correlation test was used to assess the relationship between immunological checkpoints and ENC1 expression. There must be at least one kind of cancer and an official gene symbol as inputs. When inputs are successfully provided, scatterplots that represent the statistical significance and purity-corrected partial Spearman’s rho value are created and shown. The leftmost panel is usually used to illustrate the gene expression levels versus tumor purity.

### Cell culture

We obtained endometrial cancer cell lines (HEC-1A and HEC-1B) and endometrial stromal cells (T-HESC) from the Shanghai Cell Center of the Chinese Academy of Sciences. All the cell lines were cultured in DMEM supplemented with 10% fetal bovine serum (Gibco, United States). All cell lines were cultured in a humidified 37°C incubator with 5% CO_2_.

### Quantitative real-time PCR

Total RNA was extracted using Trizol and reverse transcribed using the primescript reverse transcription reagent (Takara Bio Inc., Japan), followed by quantitative reverse transcription polymerase chain reaction (qRT-PCR) using the SYBR Green PCR kit (Takara Bio Inc., Japan) and LightCycler96 Real-time PCR apparatus (Roche). The study was performed three times, with GAPDH serving as an internal control. We utilized the following primers:

NR3C1 forward primer: 5′- ACA​GCA​TCC​CTT​TCT​CAA​CAG-3′

NR3C1 reverse primer: 5′- AGA​TCC​TTG​GCA​CCT​ATT​CCA​AT -3′

PKM2 forward primer: 5′- ATG​TCG​AAG​CCC​CAT​AGT​GAA -3′

PKM2 reverse primer: 5′- TGG​GTG​GTG​AAT​CAA​TGT​CCA -3′

ENC1 forward primer: 5′- CTT​AGA​CCT​CAC​CTA​CGT​GAC​G-3′

ENC1 reverse primer: 5′- TTG​TCC​CGG​TGC​TTG​GAT​TG -3′

GAPDH forward primer: 5′- TGT​GGG​CAT​CAA​TGG​ATT​TGG -3′

GAPDH reverse primer: 5′- ACA​CCA​TGT​ATT​CCG​GGT​CAA​T -3′

### Collection of clinical samples

Four patients who had hysterectomy at Ganzhou People’s Hospital provided endometrial cancer samples and adjacent normal tissues for study. Before surgery, these individuals did not undergo chemotherapy or radiation. This research was authorized by the hospital’s ethics committee, and patients completed informed consent forms.

### Western blotting

The RIPA reagent (Bestbio Science, Beijing, China) was used to extract protein from tissue or EC cells. The protein concentration was assayed using a BCA assay kit (Beyotime, Shanghai, China). Proteins were then separated by 10% SDS-PAGE and transferred to PVDF membranes. PVDF membranes were incubated overnight at 4°C with the primary antibodies HMGA1 (1:4,000, 5007-1-AP, Proteintech) and GAPDH (1:5,000, 60004-1-Ig, Proteintech). The membranes were then exposed for 1 hour to secondary antibodies (1:10,000, SA00001-1, SA00001-2, Proteintech). The relative intensity of the protein bands was measured with the help of ECL chemiluminescence and Image Lab analysis software. GAPDH was used as an internal reference.

## Results

### Weighted gene co-expression network analysis analysis

**FIGURE 1:**
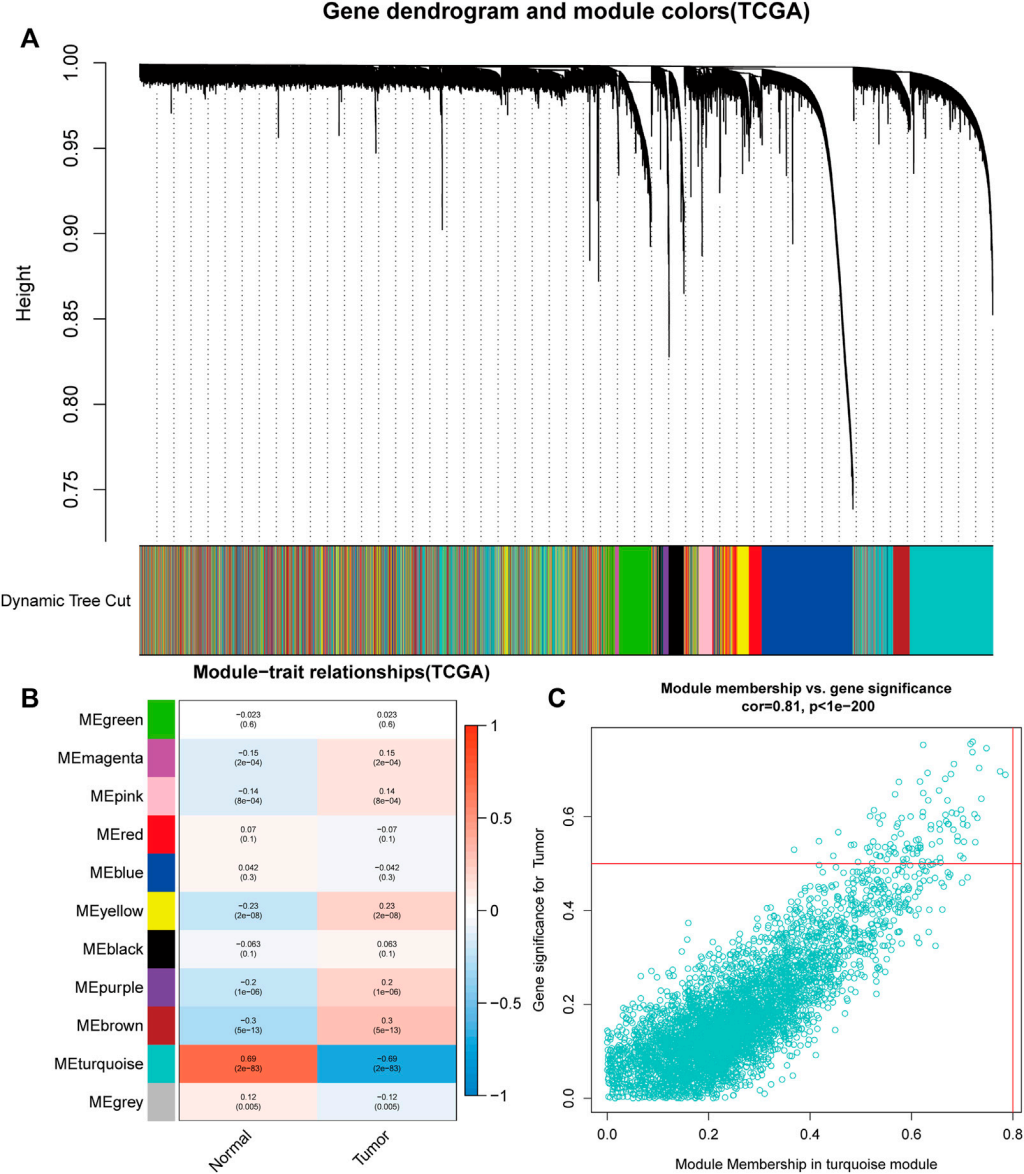
Identification of module data pertaining to the pathological tissue type in TCGA- Uterine corpus endometrial carcinoma (UCEC). **(A)** Identification of modules by dynamic tree cutting in TCGA-UCEC. **(B)** Heatmap of module–trait relationships in TCGA-UCEC. **(C)** Scatter plots of module membership vs. gene significance in the MEturquoise in TCGA-UCEC.

**FIGURE 2:**
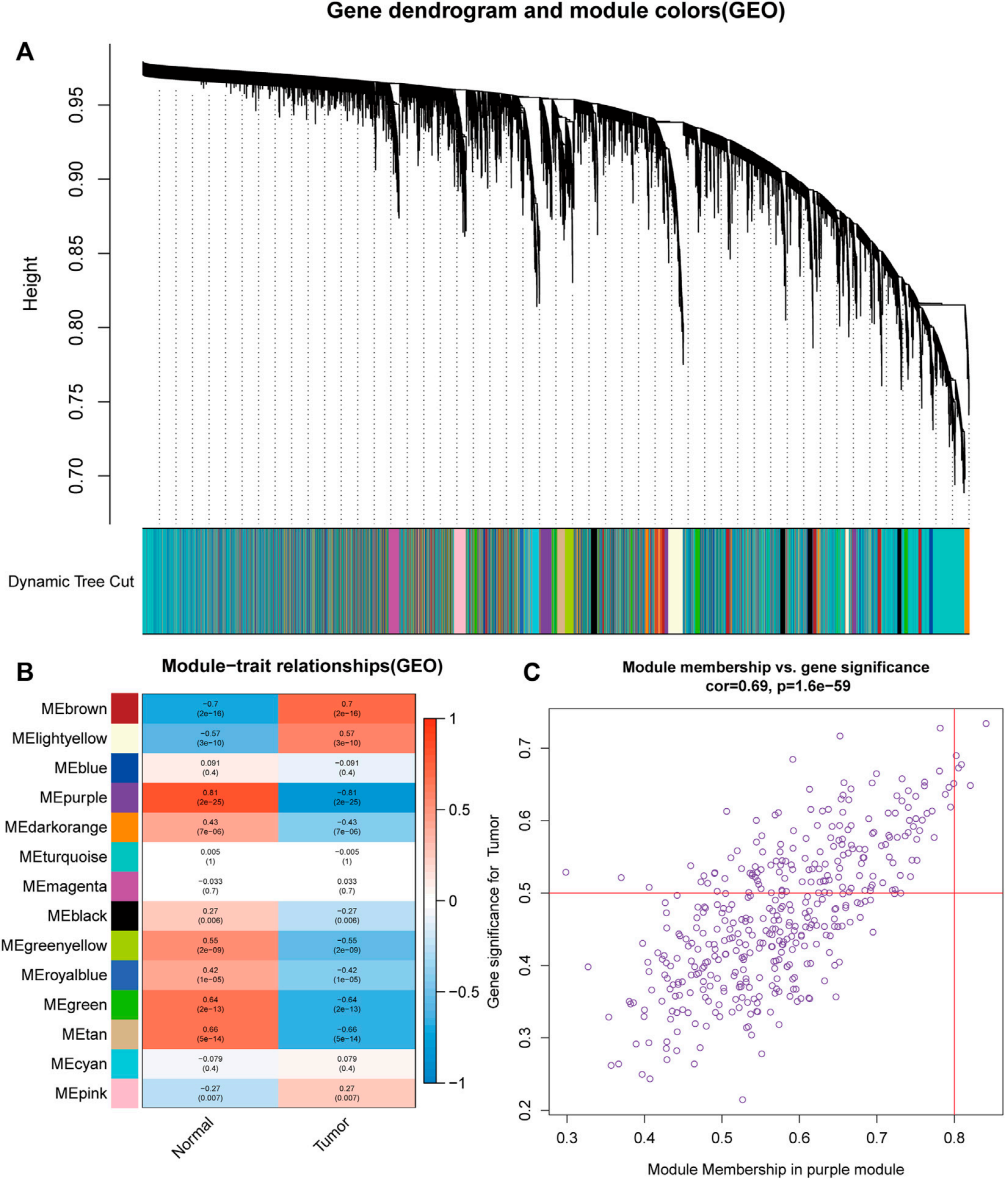
Identification of module data pertaining to the pathological tissue type in GSE17025. **(A)** Identification of modules by dynamic tree cutting in GSE17025. **(B)** Heatmap of module–trait relationships in GSE17025. **(C)** Scatter plots of module membership vs. gene significance in the MEpurple in GSE17025.

For the purpose of discovering functional gene clusters, we evaluated the TCGA-UCEC and GSE17025 datasets and built WGCNAs. We obtained an RNA-sequencing dataset from TCGA consisting of 23 normal endometrium tissue samples and 552 endometrial cancer cases with associated clinical data. Additionally, 12 normal endometrial samples and 91 endometrial cancer samples with prognostic information were retrieved from the GSE17025 dataset. Eleven TCGA-UCEC modules and fourteen GSE17025 modules were discovered in this investigation, and different colors were allocated to each module (Figure 1A, Figure 2A). After that, we looked at the relationship between each module and cancerous and non-cancerous tissue. Two modules, the MEturquoise in TCGA-UCEC (r = −0.69 p = 2e-83) and the MEpurple in GSE17025 (r = −0.81, p = 2e-25), exhibited the strongest connection with tumor tissue, according to the data (Figure 1B, Figure 2B). Correlations between module membership and gene significance for TCGA-UCEC are presented in Figure 1C (cor = 0.81, p < 1e-200), and Figure 2C illustrates the correlations for GSE17025 (cor = 0.69, *p* = 1.6 e-59).

### Acquiring DEGs and overlapping genes

**FIGURE 3:**
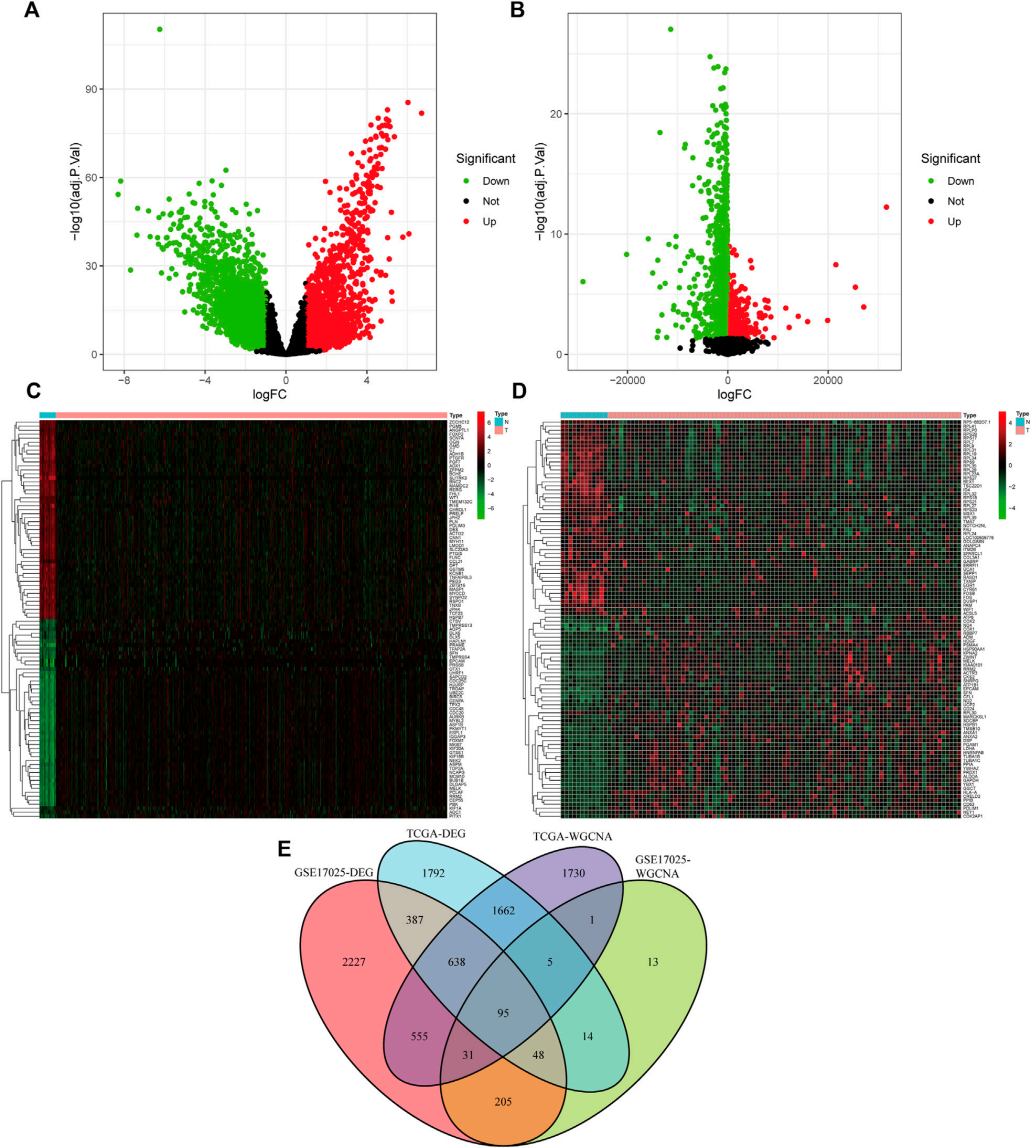
Acquiring (differentially expressed genes) DEGs and overlapping genes. **(A)** Volcano plot of the DEGs in TCGA-UCEC. **(B)**Volcano plot of the DEGs in GSE17025. **(C)** Heatmap of the DEGs in TCGA-UCEC. **(D)** Heatmap of the DEGs in GSE17025. **(E)** A Venn diagram of gene crossover between the DEGs and Weighted Gene Co-expression Network Analysis (WGCNA).

The limma software identified 4,640 (differentially expressed genes) DEGs in the TCGA dataset (Figure 3A) and 4,185 DEGs in the GSE17025 dataset (Figure 3B) as dysregulated in tumor tissues based on the cut-off criteria of |logFC| ≥ 1.0 and adj. *p* < 0.05. Among the differentially expressed genes in TCGA, we screened the top 50 genes that were up-regulated in UCEC and the top 50 genes that were down-regulated in UCEC, and we created a heat map (Figure 3C). The differentially expressed genes in GSE17025 were also shown as a heatmap using the same methodology (Figure 3D). By crossing genes in TCGA-WGCNA and GSE17025-WGCNA, as well as differentially expressed genes in TCGA-UCEC dataset and GSE17025 dataset, we obtained 95 overlapping genes. (Figure 3E).

### Assessments of functional enrichment

**FIGURE 4:**
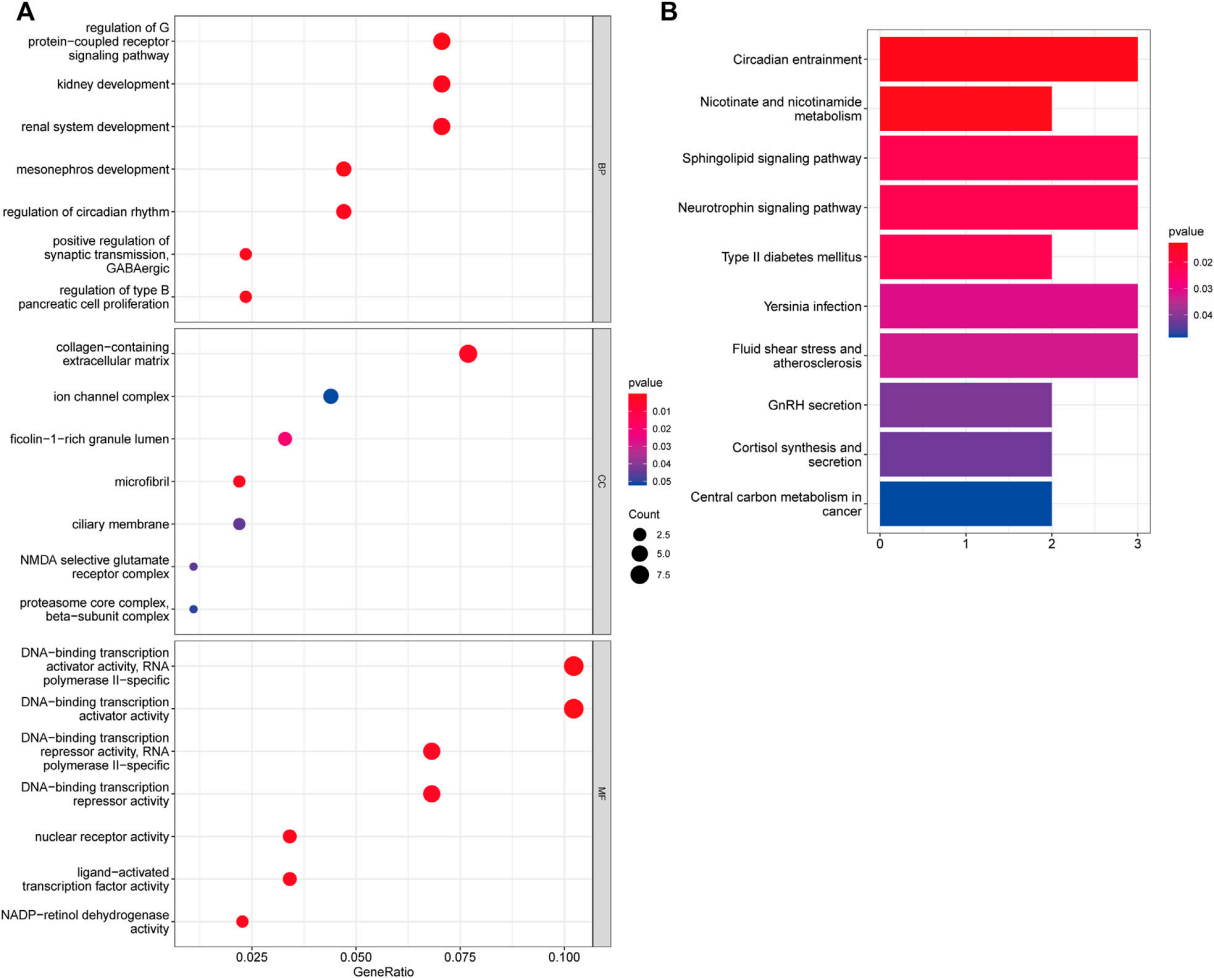
Assessments of Functional Enrichment. **(A)** Bubble plot for Gene Ontology (GO) function enrichment analysis. BP: biological processes; CC: cellular components; MF: molecular function. **(B)** Bubble plot for Kyoto Encyclopedia of Genes and Genomes (KEGG) pathway enrichment analysis. The y-axis shows pathway terms, whereas the x-axis denotes gene ratio. The size of each circle represents the number of genes. The hue of the circles symbolizes various q values.

We used the KEGG pathway and GO enrichment analyses to further investigate the function of 95 overlapped genes. Following GO enrichment analysis, it was shown that 95 overlapping genes in the biological process (BP) were mostly associated with regulation of G protein-coupled receptor signaling pathway, mesonephros development, positive regulation of synaptic transmission, and regulation of type B pancreatic cell proliferation (Figure 4A). The cellular component (CC) of 95 overlapping genes was shown to be mostly associated with microfibril, collagen-containing extracellular matrix, ficolin-1-rich granule lumen, and ciliary membrane. We discovered that the 95 overlapped genes were mostly connected to DNA-binding transcription activator activity, NADP-retinol dehydrogenase activity, nuclear receptor activity, and ligand-activated transcription factor activity using molecular function (MF) analysis. Additionally, 95 overlapping genes were associated with Circadian entrainment, Nicotinate and nicotinamide metabolism, phingolipid signaling pathway, and Neurotrophin signaling pathway in the KEGG pathway enrichment study (Figure 4B).

### Protein-protein interaction network and hub genes

**FIGURE 5:**
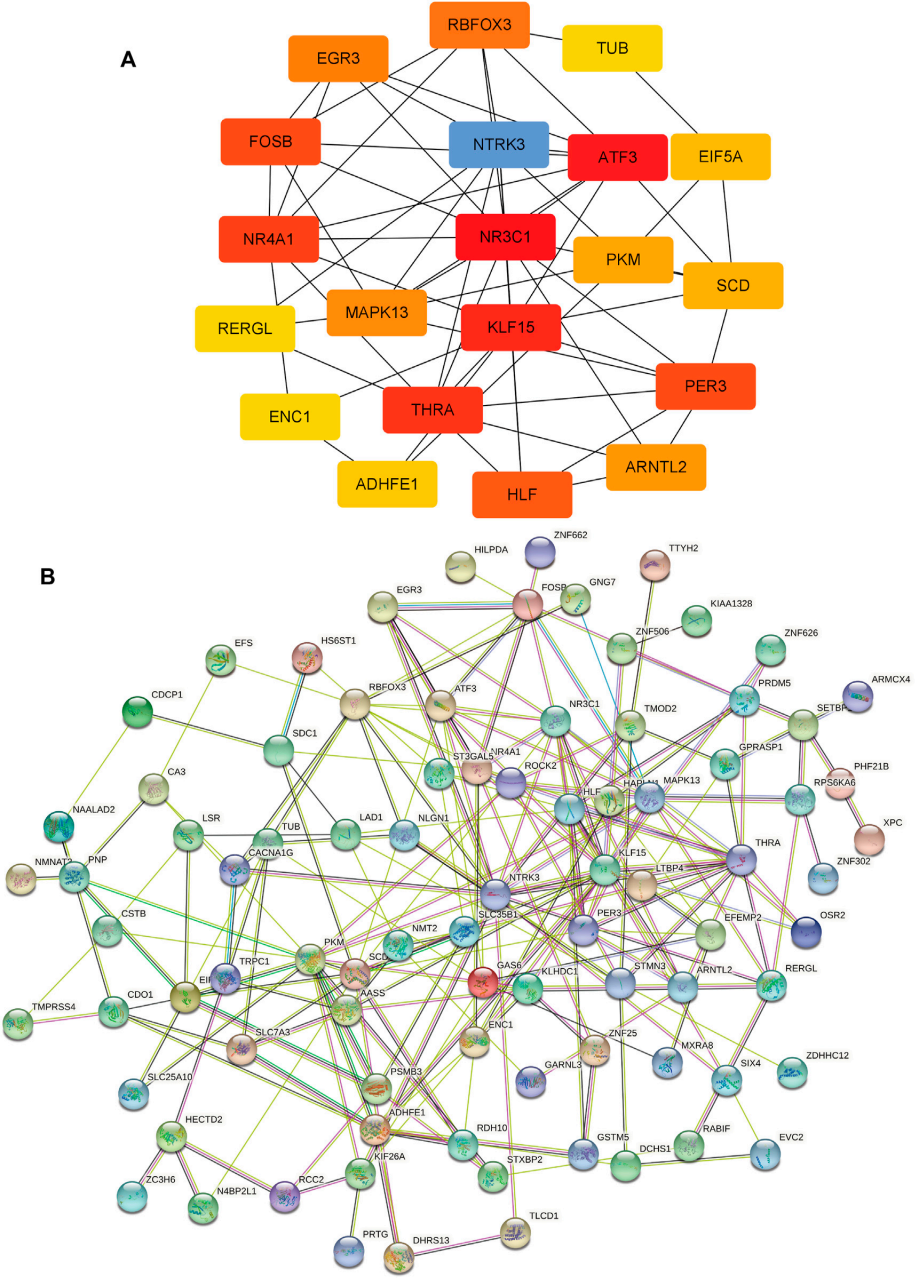
Protein-protein interaction (PPI) network and hub Genes. **(A)** 20 hub genes with the greatest Maximal Clique Centrality (MCC) value, The darker the color, the higher the MCC value. **(B)** PPI network of 95 overlapping genes.

The PPI network of 95 overlapping genes was constructed using the STRING website, and cytoscape software was used to pick the hub genes from previous analysis results (Figure 5B). The 20 genes with the greatest MCC value were identified as hub genes, including NR3C1, ATF3, KLF15, THRA, NR4A1, FOSB, PER3, HLF, NTRK3, RBFOX3, EGR3, MAPK13, ARNTL2, PKM2, SCD, EIF5A, ADHFE1, RERGL, TUB, and ENC1 (Figure 5A).

### Transcript levels of hub genes and prognosis in uterine corpus endometrial carcinoma

**FIGURE 6:**
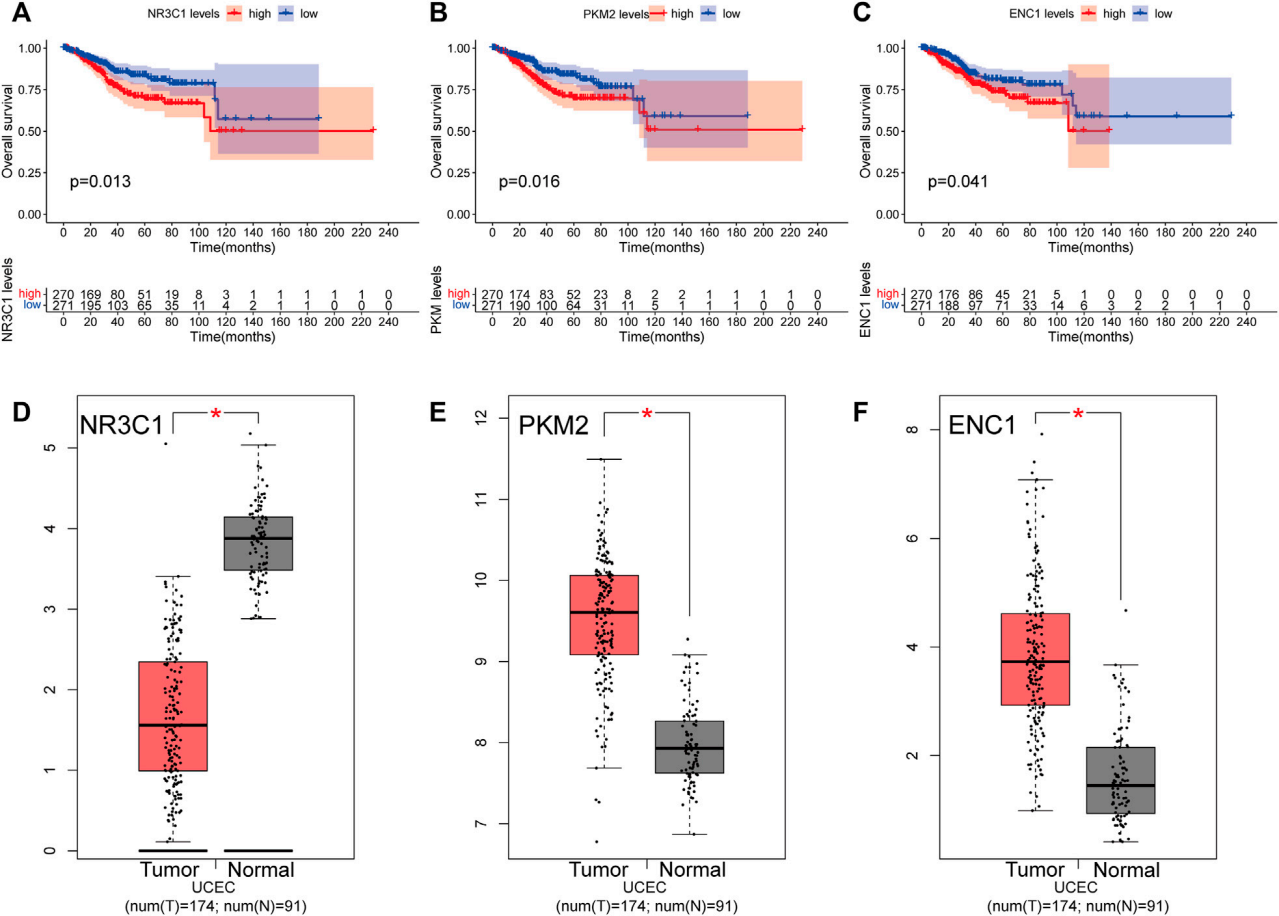
Overall survival analyses and transcript levels of genes in UCEC patients. **(A)** Overall survival analysis was performed using the Kaplan-Meier method for NR3C1 expression in UCEC patients. **(B)** Kaplan-Meier survival curves for PKM2 expression. **(C)** Kaplan-Meier survival curves for ENC1 expression **(D)** Histogram illustrating mRNA expression levels of NR3C1 in the GEPIA database. **(E)** Histogram depicting the mRNA expression levels of PKM2 in the GEPIA database. **(F)** Histogram showing the mRNA expression level of ENC1 in the GEPIA database. Red represents tumor tissue, and gray represents paracancerous tissue. *p < 0.05.

The findings indicate that the transcriptional levels of three genes, NR3C1, PKM2, and ENC1, are strongly linked with patient prognosis (Figures 6A–C). According to Kaplan–Meier analyses, increased expression of three genes was significantly related to poorer overall survival in UCEC patients (*p* < 0.05). According to the GEPIA website, PKM2 (Figure 6E) and ENC1 (Figure 6F) were over-expressed in tumor tissues and under-expressed in normal tissues, but NR3C1 (Figure 6D) was the inverse.

### Validation of hub gene expression levels by qRT-PCR and western blotting

**FIGURE 7:**
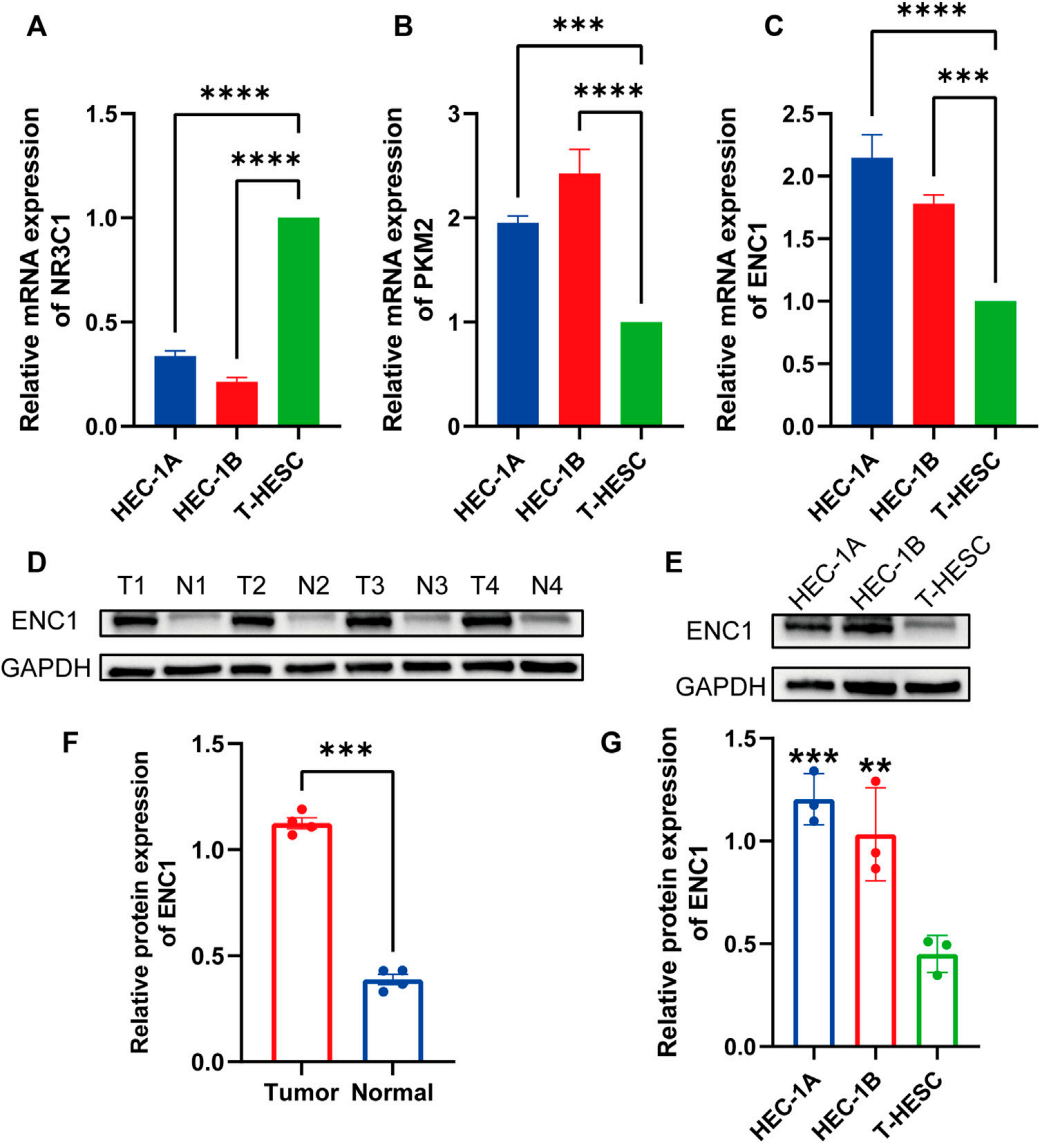
Validation of hub gene expression levels by quantitative real-time PCR and Western blotting. **(A)** The expression level of NR3C1 mRNA in endometrial cancer cell lines (HEC1A, HEC1B) was significantly lower than that in normal endometrial tissue cell lines (T-HESC). **(B)** PKM2 mRNA expression levels were significantly higher in endometrial cancer cell lines. **(C)** ENC1 mRNA expression levels were significantly higher in endometrial cancer cell lines. **(D)** Western blot analysis of ENC1 protein expression in endometrial cancer tumor and adjacent normal tissues. **(E)** Western blot analysis of ENC1 protein expression in endometrial cancer cell lines (HEC-1B and HEC1B) and normal endometrial tissue cell lines (T-HESC). **(F)** Histogram showed that the protein expression of ENC1 in endometrial carcinoma was significantly higher than that in normal tissues. **(G)** The histogram showed that the protein expression of ENC1 in two endometrial cancer cell lines was significantly higher than that in normal endometrial tissue cell lines. **p < 0.01; and ***p < 0.001; ****p < 0.0001.

To corroborate the bioinformatics findings, we used qRT-PCR to determine the mRNA expression levels of hub genes in endometrial cancer (HEC-1A, HEC-1B) and normal endometrial cell line (T-HESC). Compared to normal endometrial cell lines, the mRNA expression of NR3C1 was dramatically lowered in endometrial cancer cell lines, whereas the mRNA expression of PKM2 and ENC1 was significantly upregulated (Figures 7A–C). We discovered that the function of ENC1 in endometrial cancer has not been investigated, thus we employed western blot to investigate its expression further. The expression of ENC1 was considerably upregulated in endometrial cancer tissues compared to adjacent normal endometrial tissues, as shown by western blot analysis of clinical samples (Figures 7D,F). Similar to this, the protein expression level of ENC1 in the HEC-1A and HEC-1B cell lines was noticeably greater than that in the normal endometrial cell line (Figures 7E,G).

### The association between ENC1 and tumor immune microenvironment

**FIGURE 8:**
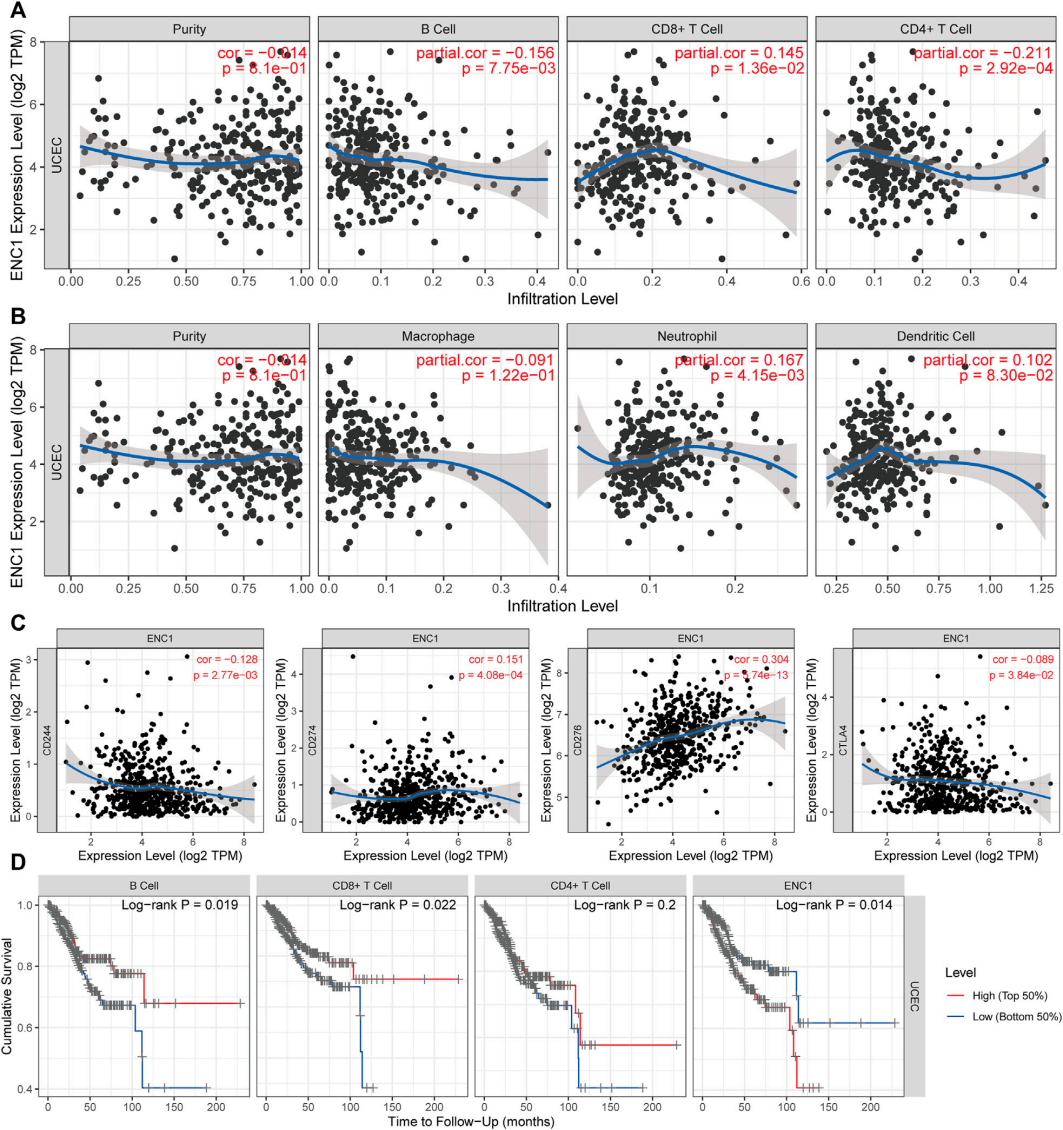
The Association between ENC1 and tumor immune microenvironment. **(A,B)** Analysis of correlations between ENC1 and immune cells. **(C)** The association between ENC1 and immune checkpoints. **(D)** Kaplan-Meier survival curves of immune cells. Patients with high B-cell infiltration or high CD8^+^ cell infiltration had a better prognosis, and those with high ENC1 expression had a worse prognosis.

Since most immune cell types have a negative correlation with tumor purity, tumor purity is a significant confounding factor in our investigation. Therefore, we choose the “Purity Adjustment” option, which would carry out this association study using the partial Spearman’s correlation. ENC1 expression was favorably connected with CD8 + T cells and Neutrophils, but negatively correlated with CD4 + T cells and B cells, according to our findings (Figures 8A,B). Immunotherapy has an interesting role to play in the treatment of cancer at the moment, and immune checkpoint blockage is an excellent technique. As a result, the link between ENC1 and immunological checkpoints was investigated (Figure 8C). Using the TIMER database, we discovered a negative link between ENC1 and a subset of immunological checkpoints (CD244 and CTLA4) but a positive correlation between ENC1 and other checkpoints (CD274 and CD276). Based on the above, we found that the expression of ENC1 in UCEC may influence the immune infiltration. Next, we investigated whether the degree of immune cell infiltration in UCEC patients correlated with prognosis (Figure 8D). Kaplan-Meier curves showed that patients with high B-cell infiltration (*p* = 0.019) or high CD8^+^ T-cell infiltration (*p* = 0.022) had a better prognosis. This suggests that immune cell infiltration in UCEC patients may be correlated with the expression level of ENC1, which needs to be further verified by experiments.

## Discussion

As a prevalent kind of gynecological malignant tumor that poses a severe danger to the health of women worldwide, the pathophysiological mechanism behind UCEC remains complex and intricate. Despite the fact that patients with endometrial cancer have a good prognosis, there are few established biochemical indicators for its identification. In our study, TCGA-UCEC and GSE17025 were used to identify genes that differed between tumor and neighboring normal tissues. It was decided to carry out more research on 95 overlapping genes. The 95 genes were then subjected to a KEGG pathway and GO enrichment analysis, which revealed that they are involved in a variety of biological activities. Additionally, the 20 genes with the greatest MCC value were identified as hub genes, including NR3C1, ATF3, KLF15, THRA, NR4A1, FOSB, PER3, HLF, NTRK3, RBFOX3, EGR3, MAPK13, ARNTL2, PKM2, SCD, EIF5A, ADHFE1, RERGL, TUB, and ENC1. According to Kaplan–Meier analyses, increased expression of three genes was significantly related to poorer overall survival in UCEC patients. According to the GEPIA website, PKM2 and ENC1 were over-expressed in tumor tissues and under-expressed in normal tissues, but NR3C1 was the inverse. To corroborate the bioinformatics findings, we used qRT-PCR to determine the mRNA expression levels of hub genes in endometrial cancer (HEC-1A, HEC-1B) and normal endometrial (T-HESC) cell lines. This research demonstrated that UCEC cell lines expressed much more PKM2 and ENC1 than control cell lines, however NR3C1 expression was decreased in these UCEC cell lines. ENC1 were also overexpressed in tumor tissues or cells, as shown by qRT-PCR and Western blotting.

Ectodermal neural cortex 1 (ENC1), also known as NRPB (nuclear restricted protein, BTB domain-like), is largely produced in the nervous system and encodes an actin binding protein (Fan et al., 2019). ENC1 regulates mutant Huntingtin aggregation and neurotoxicity through p62 under endoplasmic reticulum stress. ENC1 is controlled by the catenin/T cell factor pathway, and its dysregulation may contribute to colorectal carcinogenesis by impairing colonic cell differentiation (Lee et al., 2016). ENC1 expression is increased in human brain cancers such as glioblastomas and astrocytomas, although it is normally limited to neurons (Kim et al., 2000). Additionally, changes and mutations in the ENC1 gene may promote cell proliferation by changing nuclear cytoskeleton dynamics (Liang et al., 2004). Additionally, overexpression of ENC1 has been seen in breast carcinoma (Zhou et al., 2020; Li et al., 2021), ovarian cancer (Fan et al., 2019), lung cancer (Wu et al., 2021) and colorectal carcinoma (Cui et al., 2021b; a), suggesting that the gene may possess carcinogenic potential if overexpressed (Hammarsund et al., 2004). ENC1 expression is correlated with the transcriptional activity of the p53-regulated proteins, which explains why ENC1 is overexpressed in colorectal carcinoma (Fujita et al., 2001). However, the molecular mechanism by which ENC1 contributes to UCEC advancement and its participation in pathway cross talk remain unknown.

As is the case with other forms of cancer, genetic testing has become more significant in UCEC (Laginha et al., 2022). Endometrial immune system has unusual physiological properties, as it serves a dual purpose: it should guard against various bacteria and viruses, while also allowing implantation of an allogenic fetus (Cornish et al., 2020). To carry out these functions, estrogen and progesterone impact the endometrial microenvironment throughout the menstrual cycle (Zolton et al., 2021). ENC1 expression was favorably connected with CD8 + T cells and Neutrophils, but negatively correlated with CD4 + T cells and B cells, according to our findings. Multiple studies indicate that the existence and functionality of B cells is a significant predictive factor in cancer (Engelhard et al., 2021). Numerous malignancies have plasma cells, which are capable of producing high quantities of antibodies and cytokines. Nonetheless, tumor-infiltrating B and plasma cells possess both tumor-promoting and -suppressing properties. This is contingent on their phenotype, antibody isotype and production, tumor type, tumor microenvironment, and localization (Shalapour and Karin, 2021). Immunotherapy has an interesting role to play in the treatment of cancer at the moment, and immune checkpoint blockage is an excellent technique. Using the TIMER database, we discovered a negative link between ENC1 and a subset of immunological checkpoints (CD244 and CTLA4) but a positive correlation between ENC1 and other checkpoints (CD274 and CD276). Kaplan-Meier curves showed that patients with high B-cell infiltration (*p* = 0.019) or high CD8^+^ T-cell infiltration (*p* = 0.022) had a better prognosis. We have explored the association of ENC1 with immune infiltration based on bioinformatic analysis only, which requires a series of experiments to validate and explore the underlying mechanisms.

Immunotherapy, which entails inducing an endogenous immune response directed particularly against tumor cells, seems to be the next frontier in anticancer treatment. Numerous chemicals are available that target distinct biological pathways (Cao et al., 2021). Several of these drugs are already licensed for other cancers such as liver cancer and kidney cancer and may play a significant role in the treatment of UCEC as well (O'Malley et al., 2022; Zhao et al., 2022). These treatments are classed as active or passive: the former involves stimulating the host’s own immune system against cancer cells, whilst the latter involves administering exogenously created or modified immune system components that promote antitumor immune responses (Di Tucci et al., 2019). To circumvent the immune response, UCEC cells may activate immunological checkpoints (inhibitory mechanisms that limit T cell activation), thus engaging negative feedback processes and establishing a locally immunosuppressive environment (Saglam et al., 2022). UCEC cells have the potential to trigger programmed death-1 (PD-1) signaling by overexpressing PD-L1, which can bind to and inactivate PD-1 receptors expressed on T cells. UCEC cells overexpress PD-1 and PD-L1 to a greater extent than other types of gynecological cancer (Mutlu et al., 2022). As a result, inhibiting this route seems to be a potential method for enhancing the anticancer immune response. Pembrolizumab, a monoclonal antibody directed against PD-1, was shown to be therapeutically efficacious for the first time in a study in malignancies with DNA repair deficit (Le et al., 2015). Pembrolizumab was utilized in conjunction with Lenvatinib in 23 patients with metastatic UCEC in progression following standard chemotherapy, according to the research (Constantinidou et al., 2019). The ORR was reported to be about 50%, with modest and tolerable side effects. Nivolumab was also shown to be beneficial in two instances of recurrent ECUC that had been resistant to other therapies. Nivolumab was administered intravenously in monotherapy at a dose of 3 mg/kg semimonthly to both patients, and a substantial clinical response was noted in both instances after many months (Santin et al., 2016). CTLA4 is produced on regulatory T cells and acts as a negative immunomodulator by competitively binding ligands expressed on antigen-presenting cells, preventing them from attaching to the stimulatory receptor CD28 and therefore limiting cytotoxic T lymphocyte activation (Oh and Chae, 2019). CTLA4 is mostly responsible for controlling the activation of T cells in different parts of the body’s immune system (Mittica et al., 2017). There is compelling evidence to support the use of CTLA4 inhibitors in UCEC. However, clinical data on the effectiveness of molecules such as Ipilimumab, the anti-CTLA4 monoclonal antibodies, has not yet been published (Khalifa et al., 2022). Despite the fact that the use of CTLA4 inhibitors has a solid justification in UCEC, clinical data on the effectiveness of molecules such as Ipilimumab and Tremelimumab, two anti-CTLA4 monoclonal antibodies, have yet to be disclosed (Nishio et al., 2021; Zhang et al., 2021).

## Conclusion

We discovered three differentially expressed genes linked to UCEC prognosis using bioinformatics analysis. ENC1 were also overexpressed in UCEC tumor tissues or cell lines, as shown by quantitative real-time PCR and Western blotting. Then we looked into it further and discovered that ENC1 expression was linked to tumor microenvironment and predicted various immunological checkpoints.

## Data Availability

The datasets presented in this study can be found in online repositories. The names of the repository/repositories and accession number(s) can be found in the article/Supplementary Material.
